# Information technologies as organizational support for the COVID-19 coping actions: Nurses’ discourse

**DOI:** 10.1590/1518-8345.6202.3855

**Published:** 2023-03-27

**Authors:** Haline Costa dos Santos Guedes, José Nildo de Barros Silva, Dilyane Cabral Januário, Débora Raquel Soares Guedes Trigueiro, Oriana Deyze Correia Paiva Leadebal, Anne Jaquelyne Roque Barrêto

**Affiliations:** 1 Universidade Federal da Paraíba, João Pessoa, PB, Brazil.; 2 Scholarship holder at the Conselho Nacional de Desenvolvimento Científico e Tecnológico (CNPq), Brazil.

**Keywords:** Nursing, Nursing Informatics, Information Technology, Organizational Innovation, COVID-19, Primary Health Care, Enfermagem, Informática em Enfermagem, Tecnologia da Informação, Inovação Organizacional, COVID-19, Atenção Primária à Saúde, Enfermería, Informática en Enfermería, Tecnología de la Información, Innovación Organizacional, COVID-19, Atención Primaria de Salud

## Abstract

**Objective:**

to analyze nurses’ discourse about the potentialities in using information technologies as organizational support for the COVID-19 coping actions in Primary Health Care.

**Method:**

a qualitative and exploratory study conducted in the Family Health Strategy units from the city of João Pessoa, Paraíba, Brazil. Data collection was carried out from September to November 2021 with 26 nurses selected through the snowball technique, resorting to a semi-structured interview script. The empirical material was organized in the *Atlas.ti 9* software and grounded on the theoretical-methodological contribution of Discourse Analysis, French Line.

**Results:**

three discursive blocks were evidenced: innovation based on social media; health education actions; resoluteness in organizational actions, presenting the relevance of the WhatsApp^®^, Instagram^®^ and Facebook^®^ apps as strategic resources, in order to collaborate in the Primary Health Care are with the organization of health actions against COVID-19 by nurses.

**Conclusion:**

health units have the potential to strengthen the assistance provided through digital organizational devices; however, they need political support that invests in the structure and strategies to enhance organization of the health actions.

## Resumo

**Objetivo:**

analisar o discurso de enfermeiros acerca das potencialidades na utilização das tecnologias da informação como apoio organizacional das ações de enfrentamento da COVID-19 na Atenção Primária à Saúde.

**Método:**

estudo qualitativo e exploratório, realizado nas unidades da Estratégia Saúde da Família, na cidade de João Pessoa, Paraíba, Brasil. A coleta de dados foi realizada de setembro a novembro de 2021 com 26 enfermeiros selecionados por meio da *snowball technique*, utilizando-se de roteiro de entrevista semiestruturado. O material empírico foi organizado no software *Atlas.ti 9* e fundamentado com base no aporte teórico-metodológico da Análise do Discurso de linha francesa.

**Resultados:**

evidenciaram-se três blocos discursivos: inovação a partir das mídias sociais; ações de educação em saúde; resolutividade nas ações organizacionais, apresentando a relevância dos aplicativos WhatsApp^®^, Instagram^®^ e Facebook^®^ como recursos estratégicos, de forma a colaborar para a área de Atenção Primária à Saúde com a organização das ações de saúde frente à COVID-19 por enfermeiros.

**Conclusão:**

as unidades de saúde possuem potencial para fortalecer a assistência por meio dos dispositivos organizacionais digitais, no entanto, necessitam de apoio político que invista na estrutura e em estratégias para potencializar a organização das ações de saúde.

## Resumen

**Objetivo:**

analizar el discurso de los enfermeros sobre el potencial que tienen las tecnologías de la información como apoyo organizacional a las acciones en la lucha contra el COVID-19 en la Atención Primaria de Salud.

**Método:**

estudio cualitativo y exploratorio, realizado en unidades de la Estrategia Salud de la Familia, en la ciudad de João Pessoa, Paraíba, Brasil. La recolección de datos se realizó de septiembre a noviembre de 2021 con 26 enfermeros seleccionados mediante la técnica de bola de nieve, utilizando un formulario de entrevista semiestructurada. El material empírico fue organizado en el *software Atlas.ti 9* y se basó en el aporte teórico-metodológico del Análisis del Discurso de la escuela francesa.

**Resultados:**

se observaron tres bloques discursivos: innovación desde las redes sociales; acciones de educación para la salud; resolutividad en las acciones organizacionales, que muestran la importancia de las aplicaciones *WhatsApp* ^®^, *Instagram* ^®^ y *Facebook* ^®^ como recursos estratégicos, que colaboran en el área de Atención Primaria de Salud con la organización de las acciones de salud contra el COVID-19 que llevan a cabo los enfermeros.

**Conclusión:**

las unidades de salud tienen potencial para fortalecer la atención a través de dispositivos organizacionales digitales, pero necesitan apoyo político que invierta en la estructura y en estrategias para mejorar la organización de las acciones de salud.

**Highlights**

(1) Reflections about the organizational support provided by ITs for COVID-19 coping actions.

(2) IT as a facilitator of the health actions to cope with COVID-19, ensuring distancing.

(3) Generation of data related to IT use by nurses in the face of COVID in PHC.

## Introduction

The pandemic caused by SARS-CoV-2 represents one of the biggest health events since the 1918 Spanish flu, exerting greater effects than the HIV/AIDS pandemic in the 1980s due to its large social, economic and humanitarian proportions^1^. Up to July 5^th^, 2022, the World Health Organization (WHO) recorded 548,990,094 COVID-19 cases at the global level^2^. In the same time frame, 32,687,68 cases were confirmed in Brazil^3^.

In view of this pandemic scenario, in several countries of the world, including Brazil, centralization of the fight against the pandemic aligned with individual care in the hospital environment was observed; however, by itself, it was not sufficient to adequately and timely meet the health needs. Thus, given the need to expand the assistance provided, reorganization of the Primary Health Care (PHC) services stood out in the decentralization of mild COVID-19 cases due to the number of infected people and expansion of access for users, for considering PHC as the preferential gateway and ordering axis of the Unified Health System (*Sistema Único de Saúde*, SUS)^4^ ^-^ ^5^.

To enhance decentralization and expand the COVID-19 care scenario, the Ministry of Health (*Ministério da Saúde*, MS) encouraged Information technology (IT) use through Ordinance No. 467, dated March 20^th^ 2020^6^. IT is seen as an organizational arrangement that favors the ability to promote equality in comprehensive health care, optimize the health care praxis and help produce knowledge and expand PHC intercommunication with other care levels^7^.

On the other hand, the excess of information and news produced about COVID-19 became a concern during the pandemic. The World Health Organization (WHO) conceptualizes this situation as an infodemic, being related to excess information items, some accurate and others not, which makes it difficult to reach and locate reputable sources and reliable guidelines, favoring misinformation of the population^8^.

In this context, nurses are one of the health professionals with the potential to face the COVID-19 pandemic, for strategic action in the fight against the infodemic and effective IT use, considering the prominent position in individual and collective care practices together with the managerial activities including technical responsibility of the Health Units’ Nursing teams. However, these professionals do not always enjoy equal access to IT, which weakens the health policies targeted at COVID-19 care in PHC^9^ ^-^ ^10^.

In a literature review on IT use by nurses in coping with COVID-19, benefits were observed regarding classification of patients and sizing of the Nursing professional staff^11^, optimization of the assistance provided to patients in isolation^12^, care organization^13^ and Telenursing as a support tool for promoting maternal health^14^, all included in the hospital perspective.

It should be noted that no studies were located on the theme within the PHC scope analyzing the meanings linked to nurses’ discourse in order to justify the design and need for the current research to fill this gap in the national and international literature.

In view of the aforementioned problematization, there is an evident need to disseminate reflections regarding nurses’ role in assisting IT use and, among them, those that enhance organization of the health actions in coping with COVID-19, with an emphasis on recording diagnostic data, implementing preventive interventions, contributing to assistance actions, time optimization and qualification of the services provided.

Consequently, the research had the following question: What does nurses’ discourse about the potentialities of using ITs in COVID-19 coping management in PHC indicate? Thus, the objective of this study is to analyze nurses’ discourse about the potentialities in using information technologies as organizational support for the COVID-19 coping actions in Primary Health Care.

## Method

### Study design

A study with a qualitative approach and exploratory design, based on the theoretical-methodological contribution of Discourse Analysis (DA), French Line, supported by Michel Pêcheux’s theoretical constructs.

This analytical device proposes to understand the processes of meaning production, correlating them with the language, ideology and subject that favor understanding of the effects of meaning that language provides by and for the subjects. Without making judgments about language, DA allows an analysis through an unconscious subject, denoting the process of construction through language^15^.

During elaboration and conduction of the research, the recommendations set forth in the *Consolidated Criteria for Reporting Qualitative Research* (COREQ) were used through a checklist made available via the EQUATOR network (https://www.equator-network.org/).

### Research scenario

The research was conducted in the Family Health Strategy (FHS) units distributed across all five Health Districts (HDs) in the municipality of João Pessoa, capital city of the state of Paraíba. FHS is configured as a priority policy in the region, with 90% population coverage encompassing 211 health teams, in addition to its territorial magnitude organized in HDs that adopt IT use as support for the intervention of COVID-19 coping actions, justifying choice of this scenario. Access to the local Internet network is mostly provided by mobile data from the nurses’ own resources.

### Study participants and selection criteria

Selection of study participants was carried out using the snowball technique, also called reference chain, in order to enable non-random recognition of new participants. This technique values quality of the sample, considering the participants’ knowledge and life experiences regarding the research object, in order to reach consensus of specialized ideas^16^.

Thus, the key informant was intentionally selected: a female nurse with an effective position, who has been working in PHC for more than 20 years, with an extensive network of contacts in the field whose characteristics justified her choice. Thus, the key informant was asked to recommend new participants with the profile of individuals likely to meet the sample composition criteria, indicating their names, contacts and email addresses. The participants indicated also suggested new individuals, and so on.

Application of the theoretical saturation technique in the statements is noted, in which, through the network of contacts of 38 nurses, a continuous data analysis process was implemented during the course of the interviews, described by distancing of new elements in the discourse, resulting in the final sample of 26 participants. Consequently, the number of specialized professionals in the theme that met the study parameters was obtained, although without encompassing the total number of FHS teams from the municipality.

Selection met the following criteria: nurses with at least one year of experience in FHS in relation to COVID-19 in the local health system, and additional experience with IT in organization of the health actions in the face of COVID-19. However, nurses who were on leave and/or vacation during the collection period were excluded.

### Data collection

The data were exclusively collected between September and November 2021 by the main author, in order to minimize biases due to multiple collectors. The researcher has practical experience in PHC and in performing qualitative data collection and analysis, although she has no work or personal relationship with the study participants.

Due to the social restriction generated by the COVID-19 pandemic, previous contacts with the participants were initially established through the WhatsApp^®^ messaging app to present the project. Based on that, a pilot study was conducted aiming to evaluate, test, improve and rectify the semi-structured interview script for data collection, defined in five interviews, one in each HD of the research setting. This provided reflection and refinement of the issue, resulting in the following question: Considering your experience, could you mention the potentialities identified in the use of information technologies related to organization of the health actions in the service to face COVID-19? In addition to that, the instrument included the following sociodemographic variables: age group, gender, marital status, religion, work contract, working time, and specialization.

The interviews were scheduled beforehand according to the participants’ availability, and were conducted in a private environment as *per* each participant’s choice in the morning and afternoon shifts. The interviews were recorded using the “Voice Recorder” app on a smartphone, lasting a mean of 30 minutes, in-person, and in compliance with the biosafety measures established by the Ministry of Health regarding COVID-19.

The interviews were coded after the transcription phase, with the aid of the Google Docs text editor and using the “Speech to text” tool. In view of data reliability, all transcripts were independently reviewed by two authors, from which, after a meeting with a third author, the discursive *corpus* of the study was obtained. The participants’ anonymity was preserved, having their names represented with the acronym N, referring to the “Nursing” professional category, followed by an Arabic numeral selected at random, (e.g., N1, N2 and N26).

### Data treatment and analysis

The data were processed with the aid of the Atlas.ti software, version 9.0. This technological resource collaborates to rigor and scientificity in interpreting the meanings of the transcribed speeched, contributing to handling the data organization process. In this way, a collection of transcribed statements was generated, saved in a Word document and later hosted in the Atlas.ti software, in which appropriate quotes (codes) were created for the discursive fragments under analysis. Thus, the software exerted no influence on the delimitation of excerpts or in creating the quotes; it merely eased data organization.

The French Line of DA was adopted for data analysis, which is developed at two complementary and different moments: the analysis itself and transcription of the analysis^15^.

At the first moment, which corresponded to the analysis itself, the concept-analysis was circumscribed, also aiming to verify the analysis and saturation determined by discourse recurrence to the point of being closed. Thus, the discursive *corpus* was initially defined through floating reading, carried out without much emphasis, followed by analytical reading, in order to assist the analyst in understanding the meanings that answer the following heuristic questions: 1. Which is the concept-analysis present in the text? 2. How did the text construct the concept-analysis? 3. To which discourse does the concept-analysis constructed in the way the text builds it belong to?^15^

The concept-analysis defined in this study was “potentialities of using IT to organize the health actions to cope with COVID-19”. This concept is based on the textual signs identified in the discursive *corpus*. Subsequently, an attempt was made to identify the perceptions attributed by the nurses based on exhaustive readings, enabling recognition of the meanings linked to the textual marks identified until saturation of meanings was achieved, in order to evidence functioning of the ideology in textualization^15^.

At a second moment, the analysis was written by contextualizing and elucidating the theme, in addition to explaining the theoretical-analytical device. Thus, the theoretical assumptions that support the analysis report were understood, such as: description and interpretation related to the course of the speeches taken from the *corpus*; return of the analysis, defined by the moment in which feedback of the content that comes from the social must return to the social; and reference, concerning the annexes and appendices^15^.

### Ethical aspects

It was possible to conduct the research in PHC after obtaining approval from the Municipal Health Department and from the Research Ethics Committee of the Health Sciences Center at the Federal University of Paraíba, under protocol number 4,827,540 and Certificate of Presentation of Ethical Appreciation No. 47670621900005188. Aware of the research objectives, benefits and risks, all participants signed the Informed Consent Form, and the study was developed in compliance with all the ethical precepts set forth in Resolution No. 466/12 of the National Health Council.

## Results

The participants were 26 interviewees aged between 23 and 60 years old. There was prevalence of the female gender, with only one male representative in the final sample. Most of the nurses declared themselves as Catholic, married, with employment contracts predominantly as service providers and lasting between 1 and 35 years, and working 40 hours a week, with only one of the interviewees not having any graduate degree. 

Following the interpretation line of the meanings, it was seen that all the nurses interviewed used the WhatsApp^®^, Instagram^®^ and Facebook^®^ social media to develop health actions during the pandemic. This behavior enhanced the health and organizational actions targeted both at the users affected by the disease who were in social isolation and at those who sought the FHS units due to other health problems. In addition to that, it allowed conferring reliability to the information, guided by the WHO and the MS and minimizing the infodemic that was exponentially multiplied.

Thus, to develop such activities in the media environment, a constant connection to the Internet is necessary; however, a discursive excerpt from the speech of most of the study participants signaled that “*we have to use our mobile data to carry out these actions*”. During the pandemic, as nurses did not have Internet and a computer at their disposal, they had to develop all activities with their own resources, and some of them had difficulty handling social networks.

Based on the analysis of the participants’ statements, the following discursive blocks emerged: innovation in health actions through social media; health education actions in the face of the pandemic; and resoluteness in organizational actions.

### Innovation in health actions through social media

The testimonies indicate that the ITs used by nurses were the social media, and that these enhanced and increased capillarization of the assistance provided and organization of the health actions. Based on the discursive sequence related to the WhatsApp^®^, Instagram^®^ and Facebook^®^ codes, the following clippings stood out:

[...] *making a video, a drawing, it’s been a very good experience, it was very nice for us to make these cards and transform them, translate them and take them to the Libras language. So we were able to do this through videos and we even made a video with the employees showing the cards.* (N2)

[...] *we notify the users (send the photo via WhatsApp^®^), for example, who have COVID-19 symptoms or confirmed COVID-19 and, currently, we’re also using the vaccine app to schedule the vaccine.* (N7)

[...] *the page is Facebook ^®^ and Instagram ^®^, we have both, but we share a lot of videos through Instagram ^®^. The most accepted one is Instagram ^®^, for sure! It has many more views than Facebook ^®^.* (N11)

[...] *these patients among co-workers and us, we monitored these patients either via the phone or WhatsApp^®^ [...]* (N13)

[...] *I think I don’t even know how to work without WhatsApp^®^ with my users anymore, I don’t know how to do it anymore. I schedule cytology, prenatal, I follow-up, ask how they are, even all the notebooks, I stick, I put the phone behind, because I tell them: if you needed to, talk.* (N14)

[...] *part of WhatsApp^®^ and calls, we were even able to capture these users so that they could come and perform the test here at the unit, put a pin and see the cause, if there was going to be any worsening of the condition, it would be directed to a secondary care service [...]* (N24)

### Health education actions

Following the interpretation line of the effects of meanings, in their speeches, the nurses mentioned situations materializing IT use to develop Health Education Actions through the *professional training* and *health education* codes that were identified in the discursive block from the following excerpts:

*The patients were not lost sight of, we still have contact with these patients today, the group is still active, so they clear doubts, so it’s always a daily health action* [...] (N1)

[...] *in contrast to this part of fake news, we also saw that there was a lot of information that was accessible and that people could orient themselves through it* [...] (N8)

[...] *It organized (the Health District) doing some training sessions that took place right at the beginning and also through these remote meetings* [...] (N15)

[...] *we have a meeting, there’s helpful training, that at the time (pandemic) we were monitoring through spreadsheets too, to fill in the COVID-19 notification forms* [...] (N18)

[...] *there are classes that are online, not classes actually, training sessions that we’re also part of, that we attend regularly sometimes, we have also had online debates ... that way.* (N22)

### Resoluteness in organizational actions

In the speeches, a number of organizational devices were evidenced that optimize provision and organization of health actions through the codes of quick information, link and monitoring through which nurses assumed the strategy of communication and problem-solving through IT, as it enunciated in the lines of the following clippings:

*I think that practicality, and speed in collecting, bringing this information, this is a positive point, practical and fast, we can do it faster* [...] (N9)

[...] *the issue of communication, of facilitating that user, about us getting closer even if virtually* [...] (N10)

*Ah! It gives me access to a lot of things.... I even think it makes it easier for us to solve things, resoluteness of things, of the actions, because each health agent had their own folder so they fed it themselves and all this information.* (N16)

*Because it’s faster, right? we do everything faster, even the future electronic citizen record (PEC) will make it a lot easier for us* [...] (N26).

## Discussion

Local management acts in accordance with the MS guidance and recognizes that the monitoring of mild COVID-19 cases is a PHC responsibility, contemplating what is recommended by the SUS organizational guidelines. In this way, it guarantees organizational flow, an indispensable segment for facing COVID-19 in the municipality in question.

Faced with this challenging scenario and in order to guarantee social distancing, PHC nurses signaled in their discursive positions using WhatsApp^®^, Instagram^®^ and Facebook^®^ media tools during the pandemic, in order to organize their health actions, surfaced in their speeches and linked to Innovation through social media.

It is seen that the use of media tools is increasingly implemented, becoming a gradual practice among health professionals. A study recognizes the use of social media in the international scenario as an organizational arrangement for the dissemination and provision of health information, promotion of research projects and optimization of education for professionals and students, such as Facebook^®^, Twitter^®^, Instagram^®^ and YouTube^®^ ^17^.

WhatsApp^®^ is considered an effective device to develop Telemedicine and Telehealth; this activity was standardized for nurses in March 2020 through COFEN Resolution No. 634/2020. Thus, in the study carried out in a municipality from the Northeast, it was considered that the diverse evidence is convincing for the app to be a vehicle for health care to be used among professionals and users. A Telenursing service supported by WhatsApp^®^ in pandemic times was created and made it possible to promote health to a population vulnerable to the COVID-19 complications, as well as the development of health actions, overcoming of geographic barriers, safe welcoming and follow-up^14^.

Regarding this issue, it was evidenced that countries like India^18^, United States^19^ and Israel^20^ use WhatsApp^®^ to enable health actions that, according to a study already carried out, obtained positive feedback in relation to opinions in real time, fast communication and that information can be offered regardless of the presence of the team^14^.

Instagram^®^ was used by the nurses included the study from the perspective of organizing the health actions, developing health education activities and combating the infodemic that arose during the pandemic through more reliable information via “lives”.

In Indonesia, two studies showed that Instagram^®^ was used to monitor progression of the COVID-19 pandemic, announce restrictions and public policies, health advice or disseminate medical information and geographic spread of COVID-19, in addition to enabling the creation of public health posters targeting users with chronic diseases^21^.

Thus, for the use of this tool to be effective, it is necessary to develop strategies that encourage users’ participation, as observed in a research study^22^, which carried out a comparison between the Instagram^®^ profiles of the Portugal National Health Service and that of the Brazilian Ministry of Health, noticing non-interaction in Portugal and reduced interaction of the followers in Brazil. Thus, the need stands out to refine communication strategies aimed at increasing reach and stimulating social participation through informative texts, attractive images, hashtags adapted to the text and planning of posts^22^.

Shyly, only one female participant included in this study mentioned that the unit where she works has Facebook^®^, even though she prefers to use Instagram^®^, as she feels that the unit’s participation is more effective in it. However, the literature addresses that Facebook^®^ allows exchanging messages and that, among the social networks, it is still considered one of the most popular worldwide due to the having more than one billion users^23^.

Thus, in a state from southern Brazil, Facebook^®^ was used as a learning environment for assembling groups devoted to exchanging inputs and posts of specific contents. This reinforces that the use of innovative educational strategies enhances the development of health actions, as it provides social interaction and knowledge which, in turn, implies skills and attitudes for self-care, facilitating the users’ understanding of their role in health care^23^.

The discursive excerpts from the statements related to Health Education Actions identified in the speeches are an allusion to health education from the perspective of professional training to fight against fake news.

Health education is understood as professional or user training for the adoption of actions beneficial to collective and individual health^24^. In a state from northeastern Brazil, online transmissions were developed through Instagram^®^, without losing sight of the structuring pillars of the public policies in force in the SUS, educational topics that point to promising possibilities for continuation of the health training flow. The use of “lives” changed the ways of teaching and learning, enabled virtual communication, provided interactions in real time and at different paces between those who learn and those who teach and, in the space with greater freedom of adaptation, expanded interdisciplinarity and the contact network with physically distant bodies^25^.

Another study conducted in northeastern Brazil pointed out that nurses recognize Health Education as an important strategy for health care qualification; however, there is an obstacle regarding nurses’ interest in the pursuit of their continuous learning process, related to work overload, lack of human and material resources, and lack of motivation from the leaders^26^.

In parallel with the results pointed out by the aforementioned study, IT provides instant communication, as was observed in a study conducted in two states from southeastern Brazil, where fake news makes users more susceptible to influences, highlighting, as an example, people with low schooling levels and low family incomes. In opposition to the aforementioned study, in Portugal, a survey pointed out that parents who refused to vaccinate their children and expressed concepts of the anti-vaccination movement had university degrees and a good family incomes^27^ ^-^ ^29^. The misrepresentation caused by fake news is real and overwhelming, due to the fact that social media provide speed of dissemination, whereby the individual becomes susceptible to their interpretations, be it in a positive or negative way, and this is independent of social classes or schooling levels.

Thus, to end the discussion, the third discursive block addressing resoluteness in organizational actions was addressed, related to quick information, bonding and monitoring.

According to this study, a survey developed in the state of Paraná pointed out the ability to perform several activities concurrently and to communicate in real time as the main potentialities in IT use. This use provides efficient and constant communication between the employees, in addition to the direct relationship to achieve immediate results, which positively affects efficiency and development of the work performed. However, obstacles such as difficulty understanding, cultural differences or excess information were identified, which can distort perception of the message^30^.

The bonding addressed in this study is related to the uniqueness of people-centered attention and care. It is worth emphasizing that IT use to create a bond is a finding of this study since, in a timid way, articles dealing with this interpersonal relationship were found, although without nurses’ participation in the pandemic context.

It is seen that IT is widely used by conducting consultations through video calls in the United States, Canada, United Kingdom and China. In Brazil, online consultations since April 15^th^, 2020, have been considered as a tool that enables and enhances bonding with the users at this pandemic moment, as it provides interpersonal ties, so that users feel safe and welcomed, even if in a remote way^31^.

Successful experiences were identified in the international scenario regarding monitoring of COVID-19 cases. Among the countries are South Africa, France, Argentina, United States, South Korea, China, Australia, Italy, Cuba, India, United Kingdom and Singapore, which, although they have different PHC models, have adopted case monitoring as a prerequisite for controlling COVID-19 transmission, supported by PHC users and professionals, in order to optimize the care flow and refer to other emergency or hospital services as necessary. The United States stands out for the fact that its model of public health surveillance and health care is different and fragmented in each state, allowing for its organization to respect the autonomy of each county and federated unit^31^.

In Latin America, the National University of San Marcos, in Peru, performs Telemonitoring services for symptomatic patients and, in Argentina, the Italian Hospital of Buenos Aires develops Teleconsultations on COVID-19. In addition, the Archbishop Loayza National Hospital and the National Children’s Institute are testing Telemonitoring for patients with chronic diseases. The Latin American initiatives implied changes in the regulations to replicate successful experiences in their countries. We have Brazil as an example, which allows IT use during health emergencies due to COVID-19^32^.

More than three billion people were subjected to quarantine or social isolation. Given this scenario, digital devices are ideal for mass use because they improve accessibility to users, among other opportunities that digital tools offer, such as the search for dialogue, humanization, communication, development of health actions and comprehensiveness of care so that IT, previously little recommended, proved to be a significant solution to distancing^33^.

Social media allow connection with people, in addition to enabling interaction between users and nurses through the expression of opinions, information sharing, participation in discussions and content creation.

The current study contributes to fostering debates and reflections on IT use, in order to organize and offer health actions, for the Nursing practice and the training of nurses aimed at using IT as an essential practice in the perspective of comprehensive health care, as these technologies are capable of optimizing and qualifying the care offered to the patients, in addition to increasing productivity, improving processes and the quality of nurses’ work.

As for the limitations, it is worth noting that the research participants correspond only to a municipality with specific characteristics and training and that, for being a qualitative study, the results cannot be generalized.

## Conclusion

By considering the potentialities of information technologies as organizational support, this study described how Primary Health Care nurses use social media in their professional work during the COVID-19 pandemic, showing the relevance of WhatsApp^®^, Instagram^®^ and Facebook^®^ as strategic resources, collaborating in and facilitating organization of the health actions, with the purpose of reducing user access difficulties. The main benefits were related to innovation based on social media, health education actions and resoluteness in the organizational actions, which began to be developed through these tools only after the pandemic, mainly WhatsApp^®^, which was not employed due to the interest in preserving the work hour load and privacy of the professionals in question.

Finally, it is concluded that the implementation of social media in the performance of nurses work who make up Primary Care during the COVID-19 pandemic was positive, proving to be a strategy that enables development of the organization in health services and work processes.
